# Are we mis-estimating chemotherapy-induced peripheral neuropathy? Analysis of assessment methodologies from a prospective, multinational, longitudinal cohort study of patients receiving neurotoxic chemotherapy

**DOI:** 10.1186/s12885-019-5302-4

**Published:** 2019-02-08

**Authors:** Alex Molassiotis, Hui Lin Cheng, Violeta Lopez, Joseph S. K. Au, Alexandre Chan, Aishwarya Bandla, K. T. Leung, Y. C. Li, K. H. Wong, Lorna K. P. Suen, Choi Wan Chan, Janelle Yorke, Carole Farrell, Raghav Sundar

**Affiliations:** 1 School of Nursing, The Hong Kong Polytechnic University, Hong Kong, Hong Kong, Special Administrative Region of China; 2 Alice Lee Centre for Nursing Studies, National University of Singapore, Singapore, Singapore; 3 The Hong Kong Adventist Hospital, Hong Kong, Hong Kong, Special Administrative Region of China; 4 Department of Pharmacy, National University of Singapore, Singapore, Singapore; 5 Singapore Institute for Neurotechnology (SINAPSE), National University of Singapore, Singapore, Singapore; 6 Department of Clinical Oncology, Queen Elisabeth Hospital, Hong Kong, Hong Kong, Special Administrative Region of China; 7 Division of Nursing, Midwifery & Social Work, University of Manchester, UK and Christie NHS Foundation Trust, Manchester, UK; 8 Department of Haematology-Oncology, National University Health System, Singapore, Singapore

**Keywords:** Peripheral neuropathy, Neurotoxicity, Chemotherapy, Cancer, Assessment, Taxanes, Platins

## Abstract

**Background:**

There are inconsistencies in the literature regarding the prevalence and assessment of chemotherapy-induced peripheral neuropathy (CIPN). This study explored CIPN natural history and its characteristics in patients receiving taxane- and platinum-based chemotherapy.

**Patients and methods:**

Multi-country multisite prospective longitudinal observational study. Patients were assessed before commencing and three weekly during chemotherapy for up to six cycles, and at 6,9, and 12 months using clinician-based scales (NCI-CTCAE; WHO-CIPN criterion), objective assessments (cotton wool test;10 g monofilament); patient-reported outcome measures (FACT/GOG-Ntx; EORTC-CIPN20), and Nerve Conduction Studies.

**Results:**

In total, 343 patients were recruited in the cohort, providing 2399 observations. There was wide variation in CIPN prevalence rates using different assessments (14.2–53.4%). Prevalence of sensory neuropathy (and associated symptom profile) was also different in each type of chemotherapy, with paclitaxel (up to 63%) and oxaliplatin (up to 71.4%) showing the highest CIPN rates in most assessments and a more complex symptom profile. Peak prevalence was around the 6-month assessment (up to 71.4%). Motor neurotoxicity was common, particularly in the docetaxel subgroup (up to 22.1%; detected by NCI-CTCAE). There were relatively moderately-to-low correlations between scales (r_s_ = 0.15,*p* < 0.05-r_s_ = 0.48 *p* < 0.001), suggesting that they measure different neurotoxicity aspects from each other. Cumulative chemotherapy dose was not associated with onset and course of CIPN.

**Conclusion:**

The historical variation reported in CIPN incidence and prevalence is possibly confounded by disagreement between assessment modalities. Clinical practice should consider assessment of motor neuropathy for neurotoxic chemotherapy. Current scales may not be all appropriate to measure CIPN in a valid way, and a combination of scales are needed.

## Background

Chemotherapy- induced peripheral neuropathy (CIPN) is one of the major dose-limiting side effects of many chemotherapeutic agents including platinum analogues, vinca alkaloids, and taxanes [1]. The structure and function of peripheral motor, sensory and autonomic neurons are affected, causing peripheral neuropathic signs and symptoms [2]. In a systematic review of 31 studies (*N* = 4179), CIPN prevalence was 68.1% at the first month of chemotherapy to 30% six-months after chemotherapy, with wide variance in prevalence from 12.1–96.2%, depending on different timings of assessment and type of chemotherapy, and many assessing CIPN as part of a drug trial or with studies being cross-sectional [3].

What is already known is that neuropathic symptoms tend to progress during chemotherapy and generally regress once treatment stops; symptoms can consist of a mixture of motor, sensory, and autonomic signs; and the pain associated with CIPN can be prolonged and severe, and its treatment is usually difficult [3–5]. Furthermore, neuropathy can have a negative impact on patient’s quality of life [4]. Studies showed that CIPN is associated with fatigue, psychological distress and decline in physical independence [6–10]. CIPN may have an impact on the patients’ ability to work [11] and is associated with significantly higher medical costs ($17,344 more/patient) and higher healthcare utilization than non-CIPN patients [12].

It is clear in the literature that the assessment of CIPN is suboptimal [6]. A recent study using a Delphi technique in a small number of clinicians and consumers showed that there is no consensus as to the best assessment method for CIPN [13]. The NCI Common Toxicity Criteria (CTC) assessment for neuropathy may overestimate the presence of motor neuropathy and misdiagnose CIPN [14], although a consensus meeting suggested that the NCI CTC has good intra/interrater scores and validity [15]. Haryani et al. [16] reviewed available scales and suggested that the FACT-COG-Ntx scale and the Total Neuropathy Scale (TNS) as psychometrically optimal scales out of 20 tools examined. In another study, the Patient Neurotoxicity Questionnaire (PNQ) and the TNS were recommended [13]. The PNQ in particular seems to have received extensive evaluation in response to identified assessment problems with high variance and lack of reliability and concordance of past scales [1, 17–20], and was recommended as a patient-reported outcome preferred tool by another study [13]. It is also clear that there is a significant discrepancy between clinician-rated CIPN through available tools and patient-reported outcome scales, with clinicians under-estimating the severity of CIPN [19]. Another study showed that EORTC-CIPN20 scores may not be reliably converted to CTCAE scores [21]. While the NCI CTC score is generally considered unreliable [6], it has shown significant correlations with the more accepted and reliable TNS [22]. There are multiple studies on this topic, all providing different and often contradictory views on the most appropriate CIPN assessment method, with no ‘gold standard’ consensus being reached yet.

Longitudinal studies to systematically determine the incidence, severity and natural history of CIPN with different neurotoxic chemotherapy drugs are vital in order to quantify the extent of the problem and inform future design of interventional studies; such studies are uncommon in the literature [23]. Also, prevalence may be linked with the particular assessment scale used in each study, and the sensitivity of scales to detect CIPN is variable in the literature. While some assessment tools have received significant attention in the literature (most with variable reliability/validity issues as mentioned above), there may be other tools that can be utilised in the assessment of CIPN and have received minimal attention that are however used in other areas of medicine effectively (ie. in assessing diabetic neuropathy, etc). Hence, the present study aims to identify the natural history and progression of CIPN within different chemotherapy drugs for up to 12 months after the patient’s first infusion of chemotherapy and to analyse consistency of different assessment methods, including the introduction of some more novel approaches in CIPN assessment in detecting prevalence.

## Methods

### Design

Multinational prospective longitudinal observational cohort study over 12 months from the patient’s first neurotoxic chemotherapy infusion.

### Sample and settings

The sample included a heterogeneous group of consecutive patients receiving neurotoxic chemotherapy as inpatients or outpatients in three large hospitals in Hong Kong, Singapore, and Manchester, UK.

### Inclusion criteria

- Cancer patients who are neurotoxic chemotherapy-naïve and about to receive taxane- and/or platinum-based chemotherapy
- estimated survival of at least 12 months (as judged by the clinicians)
- aged 18+ years
- able to give written informed consent

### Sampling and procedures

Eligible patients were identified by convenient sampling and were approached at the outpatients’ clinics by a designated researcher consecutively if they met inclusion criteria. Patients were provided with detailed information about the study. Those who agreed to participate and provided signed consent completed all the baseline measurements and CIPN toxicity assessment at different time intervals. The researchers who carried out the toxicity assessments had undertaken training in neurological assessments beforehand and were provided with a set of standardized guiding questions regarding the grade of toxicity, in order to maintain consistency. Clinical data were obtained from the patients’ medical records. The study was approved by all participating hospitals and their respective ethics committees.

### Outcome measures

All assessments were carried out at baseline, at the end of each chemotherapy cycle (before or on day 1 of each chemotherapy cycle) for up to six cycles, and at 6, 9 and 12 months after enrollment into the study.

The NCI-CTCAE is a clinician-based grading system that includes criteria and definitions for quantifying the severity of CIPN in both sensory and motor components, utilizing a 5-point scale [grade 1 (asymptomatic) to grade 5 (death)]. A score of >/=2 was deemed indicative of CIPN.

The WHO-CIPN criterion is also a clinician-based grading system, which includes paresthesias, reflex decreases and extent of motor loss as parameters [24], with scores from 0 (none) to 4 (paralysis). A score of >/=1 was deemed indicative of CIPN.

Additionally, a neurological examination (deep tendon reflexes; pin sensation; strength) supplemented with a list of questions as mentioned earlier were also used to derive to the scoring of the above two scales.

Sensory examination was conducted by a research team member using: a) cotton wool to lightly touch the patient’s hands and feet bilaterally with the patients eyes closed at five points in each limb and b) 10 g monofilament test in five points in each limb which is a commonly used test in detecting diabetic neuropathy [25]. Hypo/hyperesthesia in most points touched was deemed indicative of CIPN.

Patient-reported outcome measures: For the current analysis, only the four items on numbness/tingling in hands/feet were used from the neuropathy modules of: a) The Functional assessment of cancer therapy (FACT/GOG-Ntx) [26, 27]. b) The EORTC-QLQ-C30 with its CIPN20 module [28].

Of the main study cohort, a sub-group of patients consented to nerve conduction studies (NCS) as a mode of measurement of CIPN. Each patient underwent NCS of the upper and lower limbs for assessment of neuropathy at three time points: before (NCS_baseline_), at the end of treatment (NCS_end_) and 3 months post-treatment (NCS_3m_). Sensory nerve action potential (SNAP) amplitudes and conduction velocities were measured in the bilateral medial, ulnar and radial nerves [29]. Compound motor action potential (cMAP) amplitudes and motor nerve conduction velocities were evaluated in the bilateral sural, saphenous, superficial peroneal, common peroneal and tibial nerves [30].

### Data analysis

Descriptive statistics were used to summarise the data. Prevalence estimates were calculated using 95% CIs of the percentage of patients with CIPN. Spearman correlations were used to examine the interrelationships among CTCAE-sensory, CTCAE-motor, monofilament, and WHO criterion. The dose-response relationships between cumulative chemotherapy dose and CIPN were visualized using restricted cubic splines, and the exact dose-response equations were estimated using segmented regression models. The dose-response relationship analysis was performed using R3.3.0 and the remaining statistical analysis was performed using SPSS v.23.0.

## Results

### Sample characteristics

The sample included 343 patients being assessed up to a maximum of 10 times over 12 months (total = 2399 assessments); 213 were recruited from Hong Kong, 94 from Singapore and 36 from Manchester, UK. They were at a mean age of 55.15 years old (SD = 9.4; range = 33–79). Furthermore, 33 subjects completed the NCS_baseline_ and NCS_end_ assessments, out of which 22 also completed the NCS_3m_. Among the patients who completed the NCS, 28 were female (85%), at a median age of 54 years and were receiving primarily Taxol (85%) and platinum-based chemotherapy (15%). Other overall sample characteristics are shown in Table 1.

**Table 1:** Socio-demographic and clinical characteristics of the participants (*N* = 343)

Characteristic	N	%
Sex		
Male	87	25.4
Female	256	74.6
Ethnicity		
Chinese	269	78.4
Non-Chinese Asian	31	9.0
Caucasian	43	12.5
Cancer diagnosis		
Breast cancer	174	50.7
Lung cancer	48	14.0
Gynecological cancer	46	13.4
Head & Neck cancer	30	8.7
Gastrointestinal cancer	29	8.5
Urinary tract cancer	16	4.7
Cancer stage		
I	52	15.2
II	99	28.9
III	116	33.8
IV	76	22.2
Treatment intent		
Curative	250	72.9
Definitive	30	8.7
Palliative	63	18.4
Type of chemotherapy		
Taxane	155	45.2
Platinum	109	31.8
Combination of taxane plus platinum	79	23.0
Chemotherapy protocol		
Docetaxel	122	35.6
Paclitaxel	33	9.7
Cisplatin/Carboplatin	80	23.4
Oxaliplatin	28	8.3
Carboplatin+Paclitaxel	49	14.4
Carboplatin+Docetaxel	29	8.6

### Prevalence of CIPN

**Fig. 1:**
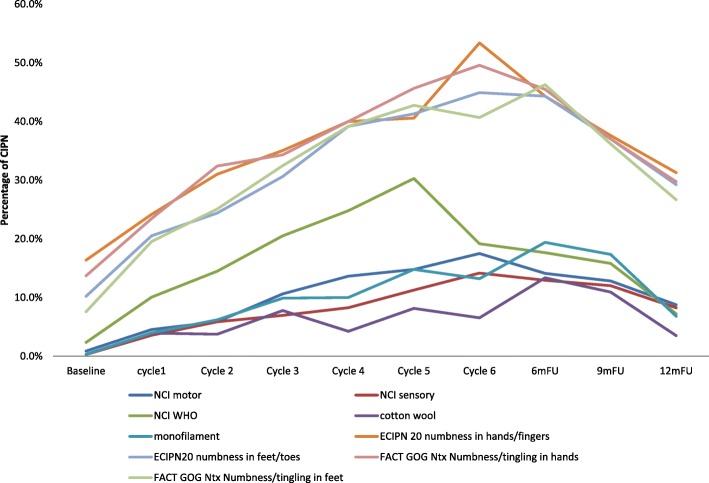
Chemotherapy-induced peripheral neuropathy prevalence by different measures over 1-year period

CIPN prevalence rates identified by different measurement tools varied significantly, and occurrence of CIPN peaked at different times. CIPN peak prevalence rates were 17.5% for the CTCAE motor criterion and 14.2% for the CTCAE sensory criterion at cycle 6; 30.3% for the WHO criterion at cycle 5; 13.4% for the cotton wool test at 6-month follow-up; 19.4% for the monofilament test at 6-month follow-up; 44.9–53.4% for the EORTC-CIPN20 items at cycle 6, and 46.3–49.6% at cycle 6 and 6-month follow-up (Table 2 and Fig. 1). Motor symptoms were slightly more frequently reported than sensory symptoms (as per CTCAE).

**Table 2:** Chemotherapy-induced peripheral neuropathy prevalence by different measures over time (*N* = 343)

Measures	Baseline		Cycle1		Cycle2		Cycle3		Cycle4		Cycle5		Cycle6		6mFU		9mFU		12mFU	
	n/N	%	n/N	%	n/N	%	n/N	%	n/N	%	n/N	%	n/N	%	n/N	%	n/N	%	n/N	%
NCI motor^1^																				
Grade 1	338/341	99.1	294/308	95.5	273/290	94.1	244/273	89.4	209/242	86.4	121/142	85.2	99/120	82.5	219/255	85.9	204/234	87.2	177/194	91.2
Grade > =2	3/341	0.9	14/308	4.5	17/290	5.9	29/273	10.6	33/242	13.6	21/142	14.8	**21/120**	**17.5**	36/255	14.1	30/234	12.8	17/194	8.8
*Grade 2*	3/341	0.9	14/308	4.5	16/290	5.5	25/273	9.2	28/242	11.6	17/142	12.0	18/120	15.0	26/255	10.2	25/234	10.7	14/194	7.2
*Grade 3*					1/290	0.4	4/273	1.5	5/242	2.1	4/142	2.8	3/120	2.5	10/255	3.9	5/234	2.1	3/194	1.6
NCI sensory^1^																				
Grade 1	340/341	99.7	297/308	96.4	273/290	94.1	254/273	93.0	222/242	91.7	126/142	88.7	103/120	85.8	222/255	87.1	205/233	88.0	178/194	91.8
Grade > =2	1/341	0.3	11/308	3.6	17/290	5.9	19/273	7.0	20/242	8.3	16/142	11.3	**17/120**	**14.2**	33/255	12.9	28/233	12.0	16/194	8.2
*Grade 2*	1/341	0.3	11/308	3.6	16/290	5.5	13/273	4.8	17/242	7.0	14/142	9.9	16/120	13.3	28/255	11.0	26/233	11.2	14/194	7.2
*Grade 3*					1/290	0.4	6/273	2.2	3/242	1.2	2/142	1.4	1/120	0.8	5/255	2.0	2/233	0.8	2/194	1.0
WHO CIPN ^2^																				
Grade 0	333/341	97.7	277/308	89.9	248/290	85.5	217/273	79.5	182/242	75.2	99/142	69.7	97/120	80.8	210/255	82.4	197/234	84.2	180/194	92.8
Grade > = 1	8/341	2.3	31/308	10.1	42/290	14.5	56/273	20.5	60/242	24.8	**43/142**	**30.3**	23/120	19.2	45/255	17.6	37/234	15.8	14/194	7.2
*Grade 1*	8/341	2.3	25/308	8.1	37/290	12.8	49/273	17.9	53/242	21.9	39/142	27.5	21/120	17.5	40/255	15.7	33/234	14.1	12/194	6.2
*Grade 2*			5/308	1.6	3/290	1.0	6/273	2.2	7/242	2.9	3/142	2.1	2/120	1.6	5/255	2.0	4/234	1.7	2/194	1.0
*Grade 3*			1/308	0.3	2/290	0.7	1/273	0.4			1/142	0.7								
Cotton wool																				
Normal (−)	306/307	99.7	268/279	96.1	257/267	96.3	237/257	92.2	203/212	95.8	124/135	91.9	100/107	93.5	188/217	86.6	163/183	89.1	138/143	96.5
Abnormal (+)	1/307	0.3	11/279	3.9	10/267	3.7	20/257	7.8	9/212	4.2	11/135	8.1	7/107	6.5	**29/217**	**13.4**	20/183	10.9	5/143	3.5
*A lot of sensation*			4/279	1.4	8/267	3.0	2/257	0.8					3/107	2.8	3/217	1.4	6/183	3.3	1/143	0.7
*A bit sensation*	1/307	0.3	1/279	0.4	1/267	0.4	2/257	0.8	2/212	0.9	1/135	0.7			21/217	9.7	12/183	6.6	4/143	2.8
*No sensation*			6/279	2.2	1/267	0.4	16/257	6.2	7/212	3.3	10/135	7.4	4/107	3.7	5/217	2.3	2/183	1.1		
Monofilament																				
Normal (−)	306/307	99.7	290/302	96.0	272/290	93.8	246/273	90.1	198/220	90.0	121/142	85.2	92/106	86.8	187/232	80.6	162/196	82.7	137/147	93.2
Abnormal (+)	1/307	0.3	12/302	4.0	18/290	6.2	27/273	9.9	22/220	10.0	21/142	14.8	14/106	13.2	**45/232**	**19.4**	34/196	17.3	10/147	6.9
*A lot of sensation*			2/302	0.7	3/290	1.0	4/273	1.5	2/220	0.9	4/142	2.8			5/232	2.2	9/196	4.6	2/147	1.4
*A bit sensation*	1/307	0.3	5/302	1.7	8/290	2.8	11/273	4.0	14/220	6.4	9/142	6.3	9/106	8.5	33/232	14.2	21/196	10.7	7/147	4.8
*No sensation*			5/302	1.7	7/290	2.4	12/273	4.4	6/220	2.7	8/142	5.6	5/106	4.7	7/232	3.0	4/196	2.1	1/147	0.7
ECIPN-20 ^3^																				
Numbness in hands/fingers																				
*Grade 1*	286/342	83.6	232/306	75.8	198/287	69.0	176/271	64.9	144/240	60.0	82/138	59.4	55/118	46.6	142/255	55.7	146/234	62.4	134/195	68.8
*Grade > =2*	56/342	16.4	74/306	24.2	89/287	31.0	95/271	35.1	96/240	40.0	56/138	40.6	**63/118**	**53.4**	113/255	44.3	88/234	37.6	61/195	31.2
*Grade 2*	50/342	14.6	55/306	18.0	62/287	21.6	72/271	26.6	66/240	27.5	36/138	26.1	36/118	30.5	73/255	28.6	67/234	28.6	49/195	25.1
*Grade 3*	5/342	1.5	12/306	3.9	21/287	7.3	16/271	5.9	21/240	8.8	14/138	10.1	19/118	16.1	27/255	10.6	16/234	6.8	9/195	4.6
*Grade 4*	1/342	0.3	7/306	2.3	6/287	2.1	7/271	2.6	9/240	3.8	6/138	4.3	8/118	6.8	13/255	5.1	5/234	2.1	3/195	1.5
Numbness in feet/toes																				
*Grade 1*	308/343	89.8	244/307	79.5	217/287	75.6	188/271	69.4	146/240	60.8	81/138	58.7	65/118	55.1	142/255	55.7	148/235	63.0	138/195	70.8
*Grade > =2*	35/343	10.2	63/307	20.5	70/287	24.4	83/271	30.6	94/240	39.2	57/138	41.3	**53/118**	**44.9**	113/255	44.3	87/235	37.0	57/195	29.2
*Grade 2*	31/343	9.0	49/307	16.0	47/287	16.4	62/271	22.9	65/240	27.1	37/138	26.8	36/118	30.5	73/255	28.6	63/235	26.8	41/195	21.0
*Grade 3*	2/343	0.6	10/307	3.3	16/287	5.6	12/271	4.4	22/240	9.2	13/138	9.4	12/118	10.2	29/255	11.4	16/235	6.8	11/195	5.6
*Grade 4*	2/343	0.6	4/307	1.3	7/287	2.4	9/271	3.3	7/240	2.9	7/138	5.1	5/118	4.2	11/255	4.3	8/235	3.4	5/195	2.6
FACTGOG-Ntx ^4^																				
Numbness/tingling in hands																				
*Grade 0*	296/343	86.3	235/307	76.5	194/287	67.6	178/271	65.7	144/240	60.0	75/138	54.3	60/119	50.4	139/255	54.5	148/235	63.0	137/195	70.3
*Grade > =1*	47/343	13.7	72/307	23.5	93/287	32.4	93/271	34.3	96/240	40.0	63/138	45.7	**59/119**	**49.6**	116/255	45.5	87/235	37.0	58/195	29.7
*Grade 1*	39/343	11.4	42/307	13.7	57/287	19.9	61/271	22.5	61/240	25.4	36/138	26.1	33/119	27.7	65/255	25.5	61/235	26.0	37/195	19.0
*Grade 2*	4/343	1.2	17/307	5.5	12/287	4.2	10/271	3.7	15/240	6.3	8/138	5.8	14/119	11.8	18/255	7.1	9/235	3.8	8/195	4.1
*Grade 3*	2/343	0.6	9/307	2.9	12/287	4.2	11/271	4.1	14/240	5.8	12/138	8.7	7/119	5.9	20/255	7.8	11/235	4.7	9/195	4.6
*Grade 4*	2/343	0.6	4/307	1.3	12/287	4.2	11/271	4.1	6/240	2.5	7/138	5.1	5/119	4.2	13/255	5.1	6/235	2.6	4/195	2.1
*Numbness/tingling in feet*																				
*Grade 0*	317/343	92.4	247/307	80.5	215/287	74.9	183/271	67.5	146/240	60.8	79/138	57.2	70/118	59.3	137/255	53.7	150/235	63.8	143/195	73.3
*Grade > =1*	26/343	7.6	60/307	19.5	72/287	25.1	88/271	32.5	94/240	39.2	59/138	42.8	48/118	40.7	**118/255**	**46.3**	85/235	36.2	52/195	26.7
*Grade 1*	17/343	5.0	35/307	11.4	44/287	15.3	58/271	21.4	65/240	27.1	35/138	25.4	30/118	25.4	69/255	27.1	54/235	23.0	34/195	17.4
*Grade 2*	6/343	1.7	12/307	3.9	10/287	3.5	9/271	3.3	13/240	5.4	5/138	3.6	9/118	7.6	22/255	8.6	13/235	5.5	7/195	3.6
*Grade 3*	2/343	0.6	7/307	2.3	10/287	3.5	11/271	4.1	10/240	4.2	13/138	9.4	6/118	5.1	17/255	6.7	9/235	3.8	9/195	4.6
*Grade 4*	1/343	0.3	6/307	2.0	8/287	2.8	10/271	3.7	6/240	2.5	6/138	4.3	3/118	2.5	10/255	3.9	9/235	3.8	2/195	1.0

^1^NCI sensory/motor grading (Grade 1 = Asymptomatic not interfering with functioning, Grade 2 = Moderate symptoms limiting instrumental ADL, Grade 3 = Severe symptom limiting self care ADL; assistive device indicated); ^2^ WHO CIPN grading (Grade 0 = None, Grade 1 = Paresthesias and/or decreased tendon reflexes, Grade 2 = severe paresthesias and/or mild weakness, Grade 3 = intolerable paresthesias and/or marked motor loss); ^3^ ECIPN-20 grading (Grade 1 = not at al, Grade 2 = a little, Grade 3 = quite a bit, Grade 4 = very much); ^4^ FACT GOG-Ntx grading (Grade 0 = not at all, Grade 1 = a little bit, Grade 2 = somewhat, Grade 3 = quite a bit, Grade 4 = very much). Bold font and underlined numbers indicate the highest prevalence of peripheral neuropathy for each measure used

**Fig. 2:**
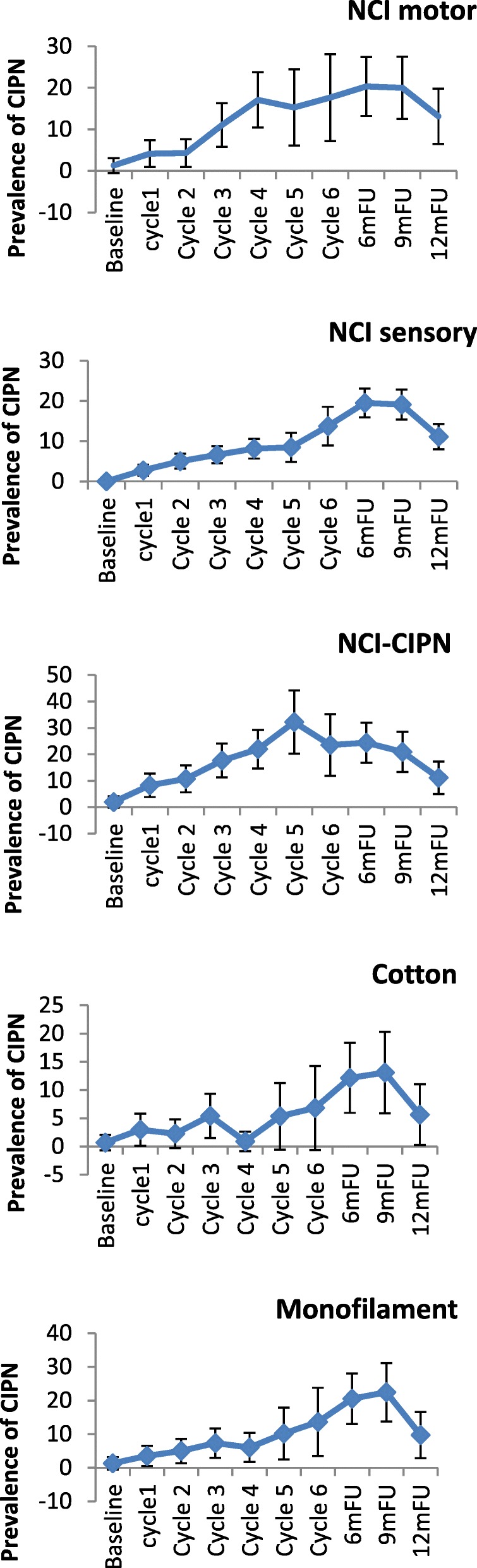

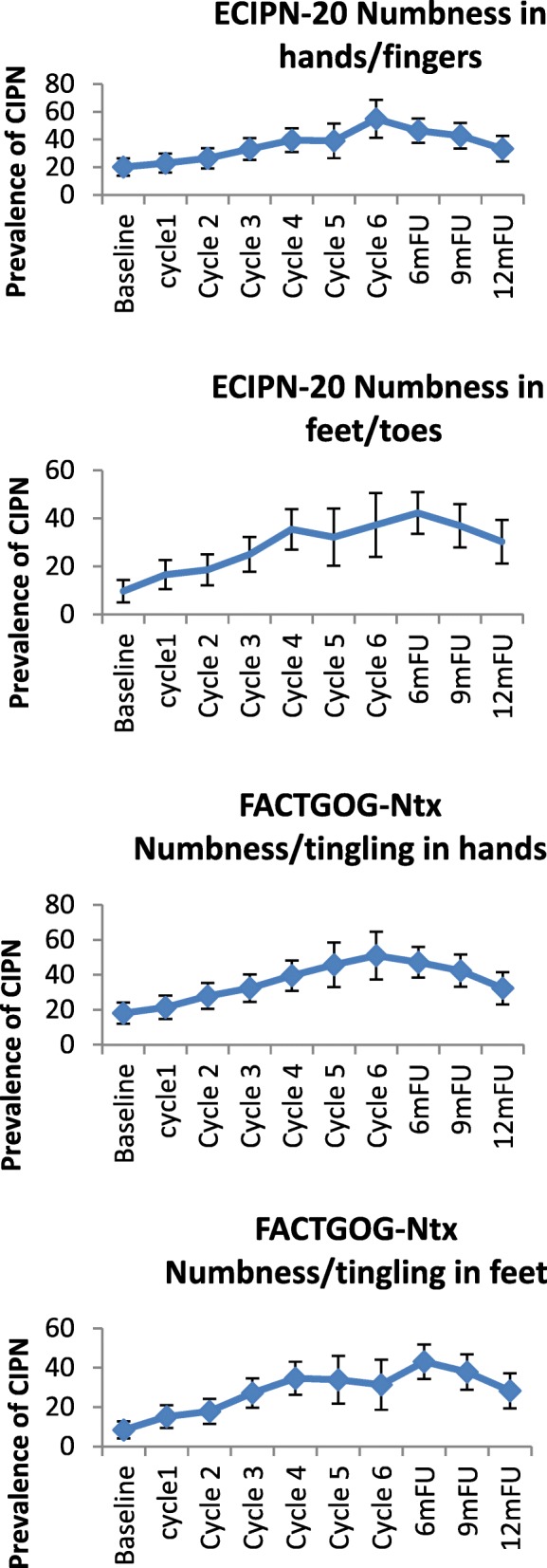
Chemotherapy-induced peripheral neuropathy (with 95% CIs) over time and with different measures used in patients receiving taxanes

**Fig. 3:**
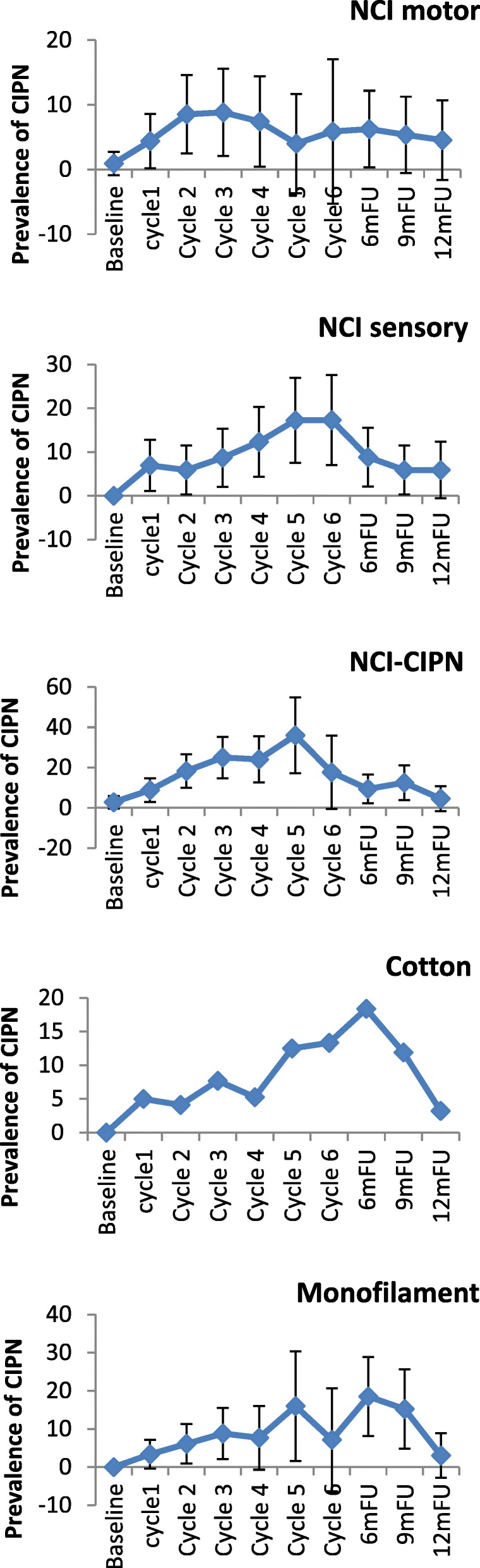

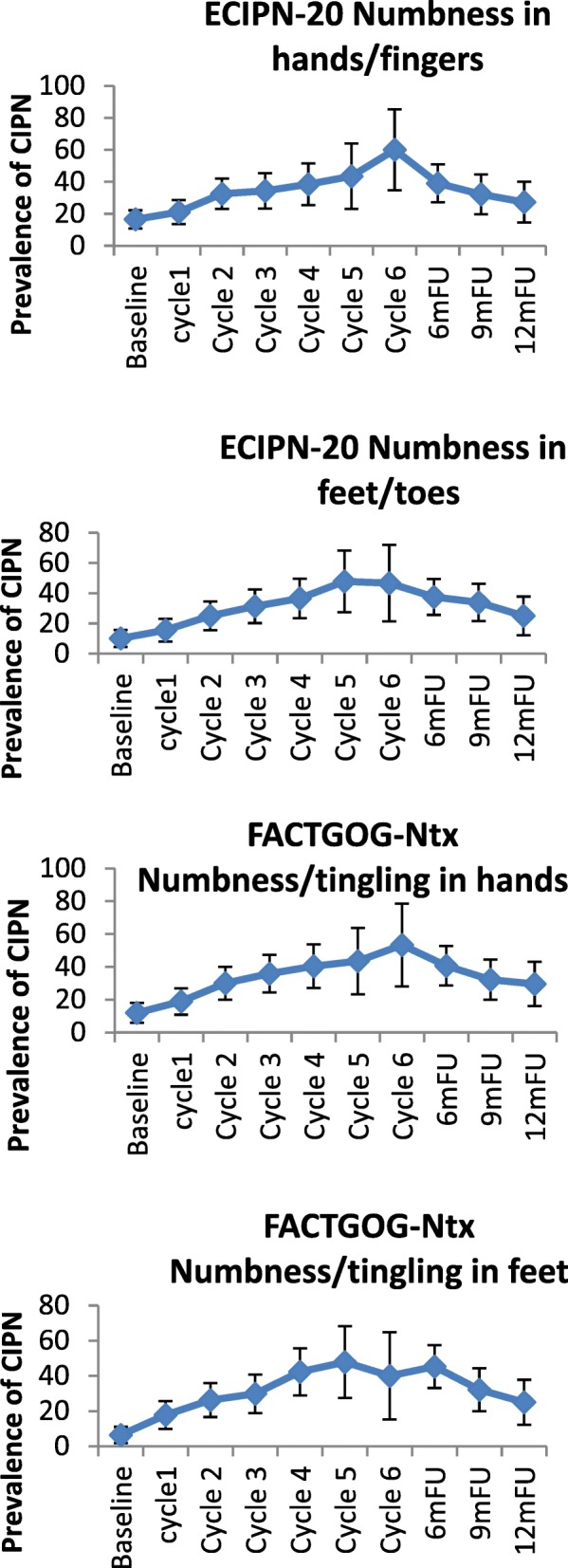
Chemotherapy-induced peripheral neuropathy (with 95% CIs) over time and with different measures used in patients receiving platinum chemotherapy

**Fig. 4:**
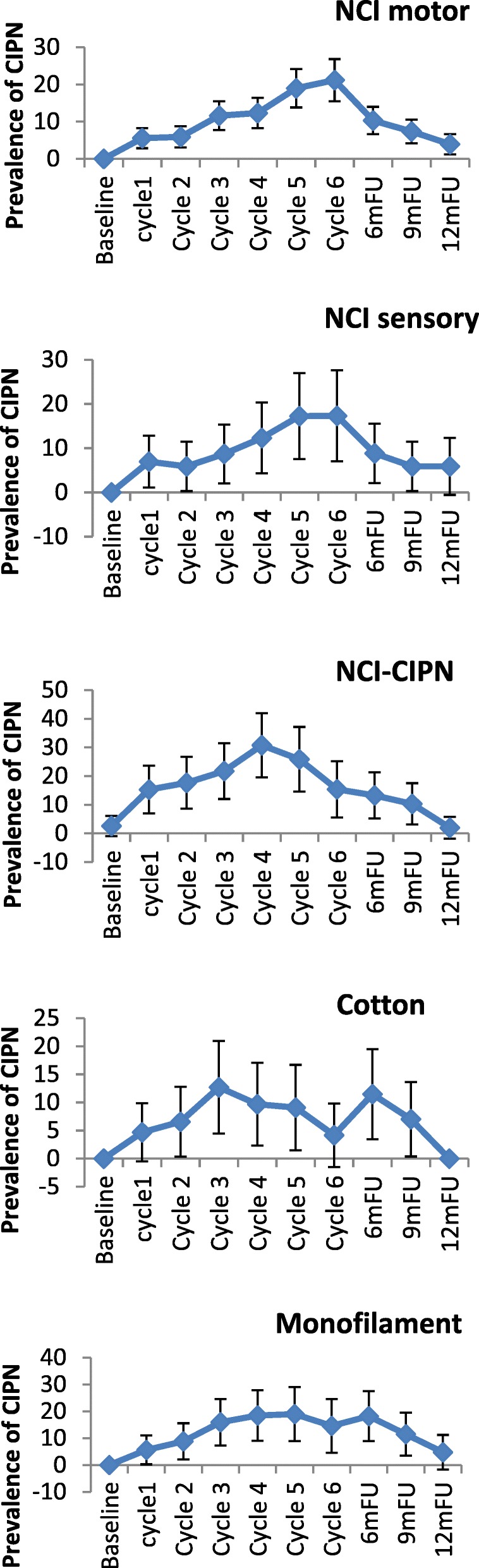

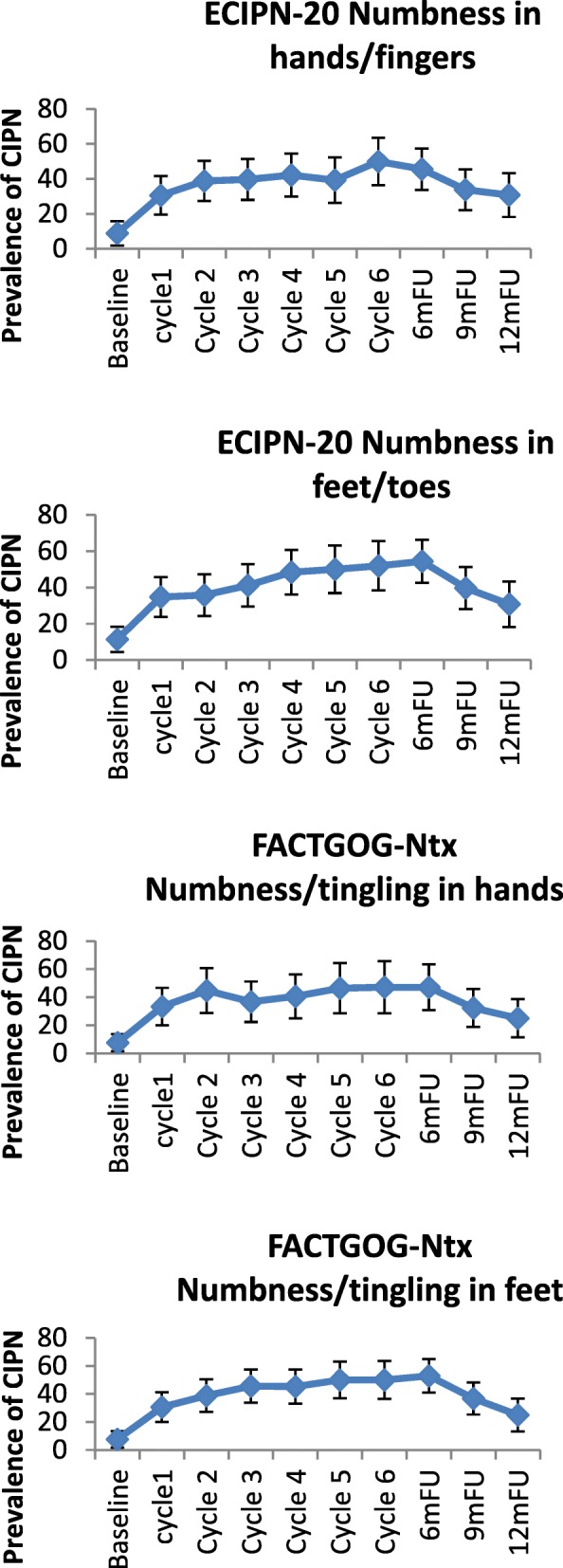
Chemotherapy-induced peripheral neuropathy (with 95% CIs) over time and with different measures used in patients receiving combination chemotherapy with taxanes and platinum

In the taxane-based group, there was a sharp increase soon into the 3rd-5th chemotherapy cycles, and rates remained high across subsequent follow-up, with only a noticeable decrease at the 12-month follow-up (Fig. 2). and peak rates ranging, depending on the scale used, from 13.1% (cotton wool; 9MFU), 19.5% (CTCAE-sensory, 6MFU), 20.3% (CTCAE-motor, 6MFU), 32.2% (WHO, cycle5), 43.1–47.2% (FACT/COG-Ntx, 6MFU) to 42.3–54.9% (EORTC, cycle 6 & 6MFU). In the platinum-based group, CIPN levels were relatively low, often being established very early (cycles 2–3) (Fig. 3). In the combination groups, noticeable increases were around cycles 3–6, decreasing significantly after the 6-month follow-up (Fig. 4). Severe CIPN scores (ie. >/=3 in CTCAE; >/=2 in WHO scale) accounted for a very small number (highest 3.9%) of patients at 6-month follow-up, although this was up to 22.9% at cycle 6 using the EORTC-CIPN20 numbness item (hands/fingers).

**Fig. 5:**
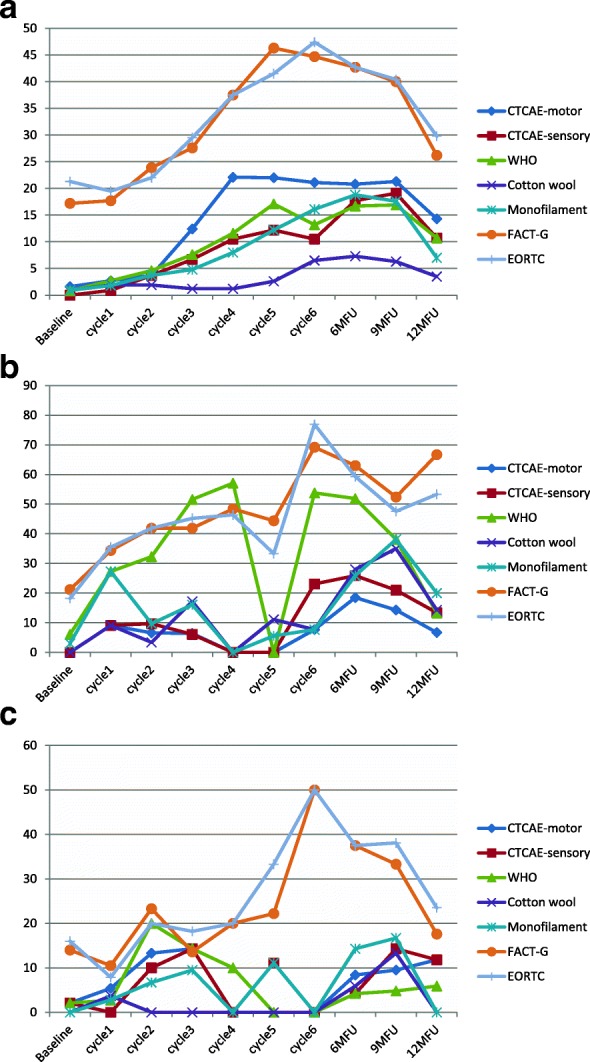

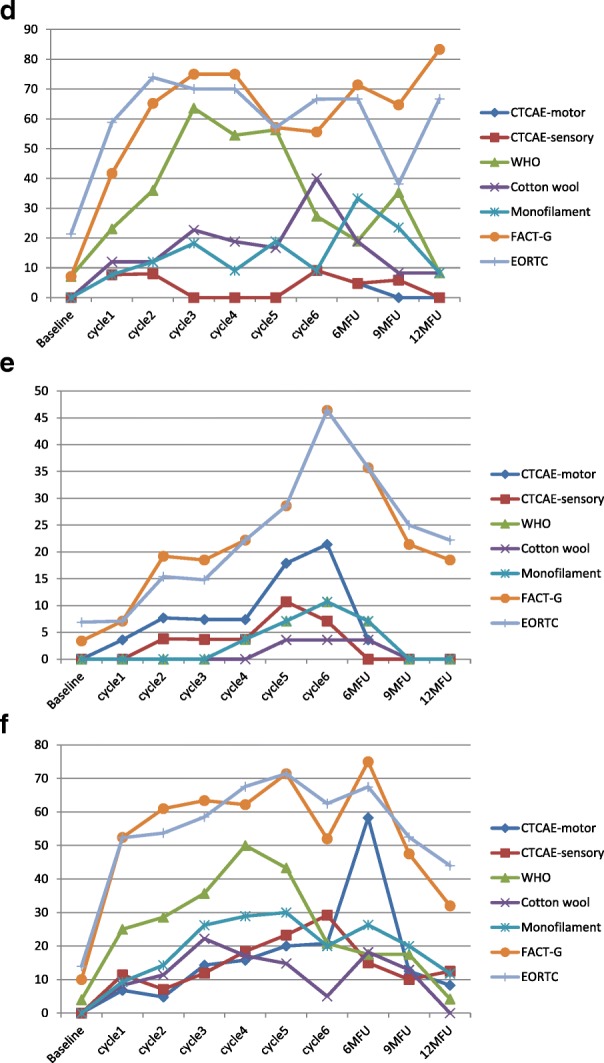
**a**. Chemotherapy-induced peripheral neuropathy prevalence over time in patients receiving Docetaxel (*n* = 122, 901 assessments) **b**. Chemotherapy-induced peripheral neuropathy prevalence over time in patients receiving Paclitaxel (*n* = 33, 245 assessments) **c**. Chemotherapy-induced peripheral neuropathy prevalence over time in patients receiving Cisplatin/Carboplatin (*n* = 80, 409 assessments) **d**. Chemotherapy-induced peripheral neuropathy prevalence over time in patients receiving Oxaliplatin (*n* = 28, 200 assessments) **e**. Chemotherapy-induced peripheral neuropathy prevalence over time in patients receiving Carboplatin+Docetaxel (*n* = 29, 274 assessments) **f**. Chemotherapy-induced peripheral neuropathy prevalence over time in patients receiving Carboplatin+Paclitaxel (*n* = 49, 357 assessments)

Within chemotherapy categories, different chemotherapy types produced differential neurotoxicity. In the taxane group, motor dysfunction was more prevalent in the docetaxel subgroup with a steady increase over cycles, with highest prevalence at 9 months (21.3%), whereas the paclitaxel subgroup showed significantly lower prevalence (Table 3 & Fig. 5a-f). The opposite was true for sensory dysfunction, where the paclitaxel subgroup had substantially higher rates of sensory problems than the docetaxel subgroup, and at times of certain assessments the difference was 5–10 times more (Table 3 & Fig. 5a-f). When paclitaxel was combined with carboplatin, again the rates of CIPN were significantly higher in this subgroup than docetaxel plus carboplatin (Table 3 & Fig. 5a-f). The prevalence varied significantly from scale to scale used for the assessment. From the clinician-based assessment (supplemented by neurological examination), highest prevalence was identified with the WHO-CIPN item followed by the use of monofilament. The patient-reported outcome measures showed significantly higher prevalence than any clinician-based measures; to note, these scales, unlike the clinician-based ones, also showed a considerable number of patients who reported symptoms indicative of CIPN at the baseline assessment (3.2–21.4%) (Table 3 & Fig. 5a-f). Looking at the PRO descriptors of CIPN (using the 9-month assessment, with all other assessments being very similar), typically patients with paclitaxel or oxaliplatin had a higher incidence than other protocols in terms of tingling in the hands/fingers (42.9 and 47.1% respectively vs 3.6–14.4%); tingling in the feet/toes (42.9 and 52.9% respectively vs 0–15.6%); burning pain in hands/feet/fingers/toes (23.8 and 23.5% respectively vs 0–9.5%); dizziness (19 and 17.6% respectively vs 3.6–8.9%); or blurred vision (38.1 and 35.3% respectively vs 10.7–27.8%). Cramps in the hands were more common in the carboplatin/cisplatin subgroup (19 and 23.5% respectively) while cramps in the feet were more common in the paclitaxel (28.6%) and oxaliplatin (29.4%) subgroup. Hearing problems were reported more often in the cisplatin/carboplatin subgroup (28.6%). Maintaining erection was reported primarily by the docetaxel subgroup (85.7%) and carboplatin/cisplatin subgroup (50%).

**Table 3:** Chemotherapy-induced peripheral neuropathy prevalence by 6 different chemotherapy drugs

CIPN measures	Samples	Baseline	Cycle1	Cycle2	Cycle3	Cycle4	Cycle5	Cycle6	6mFU	9mFU	12mFU
		n/N (%)	N (%)	N (%)	N (%)	N (%)	N (%)	N (%)	N (%)	N (%)	N (%)
NCI motor ^1^ (> = grade2)	Docetaxel	2/122 (1.6)	3/122 (2.7)	4/109 (3.7)	13/105 (12.4)	21/95 (22.1)	9/41 (22)	8/38 (21.1)	20/96 (20.8)	19/89 (21.3)	12/84 (14.3)
	Paclitaxel	0/33 (0)	3/33 (9.1)	2/31 (6.6)	2/31 (6.4)	0/28 (0)	0/13 (0)	1/13 (7.7)	5/27 (18.5)	3/21 (14.3)	1/15 (6.7)
	Cisplatin/Carboplatin	1/80 (1.2)	2/65 (3.1)	5/57 (8.8)	6/46 (13)	4/32 (12.5)	1/9 (11.1)	0/6 (0)	3/43 (7)	3/39 (7.7)	2/32 (6.2)
	Oxaliplatin	0/28 (0)	2/26 (7.7)	2/25 (8)	0/22 (0)	0/22 (0)	0/16 (0)	1/11 (9.1)	1/21 (4.8)	0/17 (0)	0/12 (0)
	Carboplatin+paclitaxel	0/49 (0)	3/44 (6.8)	2/42 (4.8)	6/42 (14.3)	6/38 (15.8)	6/30 (20)	5/24 (20.8)	14/24 (58.3)	5/40 (12.5)	2/24 (8.3)
	Carboplatin+docetaxel	0/29 (0)	1/28 (3.6)	2/26 (7.7)	2/27 (7.4)	2/27 (7.4)	5/26 (17.9)	6/28 (21.4)	1/28 (3.6)	0/28 (0)	0/27 (0)
NCI sensory^1^ (> = grade2)	Docetaxel	0/122 (0)	1/122 (0.9)	4/109 (3.7)	7/105 (6.7)	10/95 (10.5)	5/41 (12.2)	4/38 (10.5)	17/96 (17.7)	17/89 (19.1)	9/84 (10.7)
	Paclitaxel	0/33 (0)	3/33 (9.1)	3/31 (9.7)	2/31 (6.14)	0/28 (0)	0/18 (0)	3/13 (23.1)	7/27 (25.9)	4/21 (19)	2/15 (13.4)
	Cisplatin/Carboplatin	1/80 (1.2)	0/65 (0)	4/57 (7.0)	4/46 (8.7)	2/32 (6.3)	1/9 (11.1)	0/6 (0)	2/43 (4.7)	3/39 (7.7)	2/32 (6.2)
	Oxaliplatin	0/28 (0)	2/26 (7.7)	2/25 (8)	0/22 (0)	0/22 (0)	0/16 (0)	1/11 (9.1)	1/21 (4.8)	1/17 (5.9)	0/12 (0)
	Carboplatin+paclitaxel	0/49 (0)	5/44 (11.4)	3/42 (7.1)	5/42 (11.9)	7/38 (18.4)	7/30 (23.3)	7/27 (29.2)	6/40 (15)	4/40 (10)	3/24 (12.5)
	Carboplatin+docetaxel	0/29 (0)	0/28 (0)	1/26 (3.8)	1/27 (3.7)	1/27 (3.7)	3/28 (10.7)	2/28 (7.1)	0/28 (0)	0/28 (0)	0/27 (0)
WHO-CIPN ^2^ (> = grade 1)	Docetaxel	1/122 (0.8)	3/122 (2.7)	5/109 (4.6)	8/105 (7.6)	11/95 (11.6)	7/41 (17.1)	5/38 (13.2)	16/96 (16.7)	15/89 (16.9)	9/84 (10.7)
	Paclitaxel	2/33 (6.1)	9/33 (27.3)	10/31 (32.3)	16/31 (51.6)	16/28 (57.1)	0/18 (0)	7/13 (53.8)	14/27 (51.9)	8/21 (38.1)	2/15 (13.3)
	Cisplatin/Carboplatin	1/80 (1.2)	2/65 (3)	6/57 (10.5)	3/46 (6.5)	1/32 (3.1)	0/9 (0)	0/6 (0)	2/43 (4.7)	1/39 (2.6)	1/32 (3.1)
	Oxaliplatin	2/28 (7.1)	6/26 (23.1)	9/25 (36)	14/22 (63.6)	12/22 (54.5)	9/16 (56.3)	3/11 (27.3)	4/21 (19)	6/17 (35.3)	1/12 (8.3)
	Carboplatin+paclitaxel	2/49 (4)	11/44 (25)	12/42 (28.6)	15/42 (35.7)	19/38 (50)	13/30 (43.3)	5/19 (20.8)	7/40 (17.5)	7/40 (17.5)	1/24 (4.2)
	Carboplatin+docetaxel	0/29 (0)	0/28 (0)	0/26 (0)	0/27 (0)	1/27 (3.7)	2/28 (7.1)	3/28 (10.7)	2/28 (7.1)	0/28 (0)	0/27 (0)
Cotton wool(+)^3^	Docetaxel	1/113 (0.9)	2/104 (1.9)	2/104 (1.9)	2/100 (1.2)	1/84 (1.2)	1/38 (2.6)	2/31 (6.5)	6/82 (7.3)	4/64 (6.3)	2/57 (3.5)
	Paclitaxel	0/31 (0)	3/33 (9.1)	1/29 (3.4)	5/29 (17.2)	0/28 (0)	2/18 (11.1)	1/13 (7.7)	7/25 (28)	7/20 (35)	2/14 (14.3)
	Cisplatin/Carboplatin	0/65 (0)	1/55 (1.8)	0/48 (0.0)	0/43 (0)	0/16 (0)	0/8 (0)	0/3 (0)	1/29 (3.4)	2/26 (7.7)	0/19 0)
	Oxaliplatin	0/27 (0)	3/25 (12.0)	3/25 (12)	5/22 (22.7)	3/16 (18.8)	2/12 (16.7)	8/20 (40)	3/16 (18.8)	1/12 (8.3)	1/12 (8.3)
	Carboplatin+paclitaxel	0/49 (0)	6/36 (8.3)	4/35 (11.4)	8/36 (22.2)	6/29 (17.1)	4/27 (14.8)	1/20 (5)	6/33 (18.2)	4/31 (12.9)	0/16 (0)
	Carboplatin+docetaxel	0/29 (0)	0/28 (0)	0/26 (0)	0/27 (0)	0/27 (0)	1/28 (3.6)	1/28 (3.6)	1/28 (3.6)	0/26 (0)	0/25 (0)
Monofilame-nt(+)^3^	Docetaxel	1/116 (0.9)	2/109 (1.8)	4/109 (3.7)	5/105 (4.8)	7/88 (8)	5/41 (12.2)	5/31 (16.1)	16/85 (18.8)	12/68 (17.6)	4/57 (7.0)
	Paclitaxel	1/33 (3.0)	9/33 (27.3)	3/31 (9.7)	5/31 (16.1)	0/31 (0)	1/18 (5.6)	1/13 (7.7)	7/27 (25.9)	8/21 (38.1)	3/15 (20.0)
	Cisplatin/Carboplatin	0/77 (0)	1/63 (1.6)	2/57 (3.5)	2/46 (4.3)	1/17 (5.9)	1/9 (0)	0/3 (0)	3/33 (9.1)	3/29 (10.3)	0/21 (0)
	Oxaliplatin	0/28 (0)	2/26 (7.7)	3/25 (12)	4/22 (18.2)	2/22 (9.1)	3/16 (18.8)	1/11 (9.1)	7/21 (33.3)	4/17 (23.5)	1/12 (8.3)
	Carboplatin+paclitaxel	0/49 (0)	4/43 (9.3)	6/42 (14.3)	11/42 (26.2)	11/38 (28.9)	9/30 (30)	4/20 (20)	10/38 (26.3)	7/35 (20)	2/17 (11.8)
	Carboplatin+docetaxel	0/29 (0)	0/28 (0)	0/26 (0)	0/27 (0)	1/27 (3.7)	2/28 (7.1)	3/28 (10.7)	2/28 (7.1)	0/26 (0)	0/25 (0)
FACTGOG-Ntx Numbness/tingling in hands ^5^ (>grade = 1)	Docetaxel	21/122 (17.2)	20/113 (17.7)	26/109 (23.9)	29/105 (27.6)	36/96 (37.5)	19/41 (46.3)	17/38 (44.7)	41/96 (42.7)	36/90 (40)	22/84 (26.2)
	Paclitaxel	7/33 (21.2)	11/32 (34.4)	13/31 (41.9)	15/31 (41.9)	13/28 (48.4)	8/18 (44.4)	9/13 (69.2)	17/27 (63)	11/21 (52.4)	10/15 (66.7)
	Cisplatin/Carboplatin	11/81 (13.6)	7/66 (10.6)	9/57 (15.8)	9/47 (19.1)	6/32 (18.7)	2/9 (22.2)	3/6 (50)	11/43 (25.6)	7/39 (17.9)	3/32 (9.4)
	Oxaliplatin	2/28 (7.1)	10/24 (41.7)	15/26 (65.2)	15/20 (75)	15/20 (75)	8/14 (57.1)	5/9 (55.6)	15/21 (71.4)	11/17 (64.7)	10/12 (83.3)
	Carboplatin+paclitaxel	5/50 (10)	23/44 (52.4)	25/41 (61.0)	22/41 (53.7)	22/37 (59.5)	18/28 (64.3)	13/25 (52)	22/40 (55)	16/40 (40)	8/25 (32)
	Carboplatin+docetaxel	1/29 (3.4)	1/28 (3.6)	5/26 (19.2)	3/27 (11.1)	4/27 (14.8)	8/28 (28.6)	12/28 (42.9)	10/28 (35.7)	6/28 (21.4)	5/27 (18.5)
FACTGOG-Ntx Numbness/tingling in feet ^5^ (>grade = 1)	Docetaxel	6/122 (4.9)	10/113 (8.8)	12/109 (11)	22/105 (21)	29/96 (30.2)	12/41 (29.3)	9/38 (23.7)	37/96 (38.5)	31/90 (34.4)	21/84 (25.0)
	Paclitaxel	5/32 (15.6)	11/31 (35.5)	13/31 (41.9)	15/31 (8.4)	14/28 (50)	8/18 (44.4)	7/13 (53.8)	16/27 (59.3)	10/21 (47.6)	7/15 (46.7)
	Cisplatin/Carboplatin	5/81 (6.2)	7/66 (10.6)	5/57 (8.8)	7/47 (14.9)	7/32 (21.9)	3/9 (33.3)	3/6 (50)	14/43 (32.6)	8/39 (20.5)	2/32 (6.2)
	Oxaliplatin	2/28 (7.2)	9/27 (37.5)	16/23 (69.6)	13/20 (65)	15/20 (75)	8/14 (57.1)	3/9 (33.3)	15/21 (71.4)	5/21 (23.8)	9/12 (75.0)
	Carboplatin+paclitaxel	5/50 (10)	20/44 (45.5)	22/41 (53.7)	26/41 (63.4)	23/37 (62.2)	20/28 (71.4)	13/25 (52)	26/40 (75)	19/40 (47.5)	8/25 (32)
	Carboplatin+docetaxel	1/29 (3.4)	2/28 (7.1)	4/26 (15.4)	5/27 (18.5)	6/27 (22.2)	8/28 (28.6)	13/28 (46.4)	10/28 (35.7)	6/28 (21.4)	5/27 (18.5)
ECIPN-20 (item 3) Numbness inhands/fingers ^4^ (> = grade 2)	Docetaxel	26/122 (21.3)	22/113 (19.5)	24/109 (22)	31/105 (29.5)	36/96 (37.5)	17/41 (41.5)	18/38 (47.4)	41/96 (42.7)	36/89 (40.4)	25/84 (29.8)
	Paclitaxel	6/33 (18.2)	11/31 (35.5)	13/31 (41.9)	14/31 (45.2)	13/28 (46.4)	6/18 (33.3)	10/13 (76.9)	10/27 (59.3)	10/21 (47.6)	8/15 (53.3)
	Cisplatin/Carboplatin	12/81 (14.8)	5/66 (7.6)	9/57 (15.8)	9/47 (19.1)	6/32 (18.7)	3/9 (33.3)	3/6 (50)	11/43 25.6)	8/39 (20.5)	4/32 (22.5)
	Oxaliplatin	6/28 (21.4)	14/24 (58.8)	17/23 (73.9)	14/20 (70.0)	14/20 (70.0)	8/14 (57.1)	6/9 (66.6)	14/21 (66.7)	10/17 (38.1)	8/12 (66.7)
	Carboplatin+paclitaxel	5/50 (10)	20/44 (45.5)	22/41 (53.7)	23/41 (56.1)	23/37 (62.2)	15/28 (53.6)	13/24 (54.2)	22/40 (55)	16/40 (40)	10/25 (40)
	Carboplatin+docetaxel	2/29 (6.9)	2/28 (7.1)	4/26 (15.4)	4/27 (14.8)	4/27 (14.8)	7/28 (25)	11/28 (39.3)	9/28 (32.1)	7/28 (25)	6/27 (22.2)
ECIPN-20 (item 4) Numbness in feet/toes ^4^ (> = grade 2)	Docetaxel	8/122 (6.6)	12/113 (10.6)	15/109 (13.8)	23/105 (21.9)	30/96 (31.2)	10/41 (24.4)	11/38 (29.0)	37/96 (38.5)	31/90 (34.4)	24/84 (28.6)
	Paclitaxel	7/33 (21.2)	12/32 (37.5)	11/31 (35.5)	11/31 (35.5)	14/28 (50.0)	9/18 (50.0)	8/13 (61.5)	15/27 (55.6)		6/15 (40)
	Cisplatin/Carboplatin	8/81 (9.9)	4/66 (6.1)	6/57 (10.5)	7/47 (14.9)	5/32 (15.6)	3/9 (33.3)	3/6 (50)	11/43 (25.6)	9/39 (23.1)	3/32 (9.4)
	Oxaliplatin	3/28 (10.7)	10/24 (41.7)	14/9 (60.1)	14/20 (70.0)	14/20 (70)	7/14 (50.0)	4/9 (44.4)	13/21 (61.9)	10/17 (58.8)	8/12 (66.7)
	Carboplatin+paclitaxel	7/50 (14)	23/44 (52.3)	21/41951.2)	24/41 (58.5)	25/37 (67.6)	20/28 (71.4)	15/24 (62.5)	27/40 (67.5)	21/40 (52.5)	11/25 (440
	Carboplatin+docetaxel	2/29 (6.9)	2/28 (7.1)	3/26 (11.5)	4/27 (14.8)	6/27 (22.2)	8/28 (28.6)	13/28 (46.4)	10/28 (35.7)	6/28 (21.4)	5/27 (18.5)

^1^NCI motor/sensory (>grade 2 = Moderate symptoms limiting instrumental ADL); ^2^ WHO CIPN (> = Grade 1 = Paresthesias and/or decreased tendon reflexes); ^3^ cotton wool/monofilament (+) = (abnormal sensation, a lot/a little/no sensation); ^4^ ECIPN-20 (> = grade 2 = A little); ^5^ FACTGOG-Ntx (> = 1 = a little bit)

### Cumulative chemotherapy dose and CIPN

**Fig. 6:**
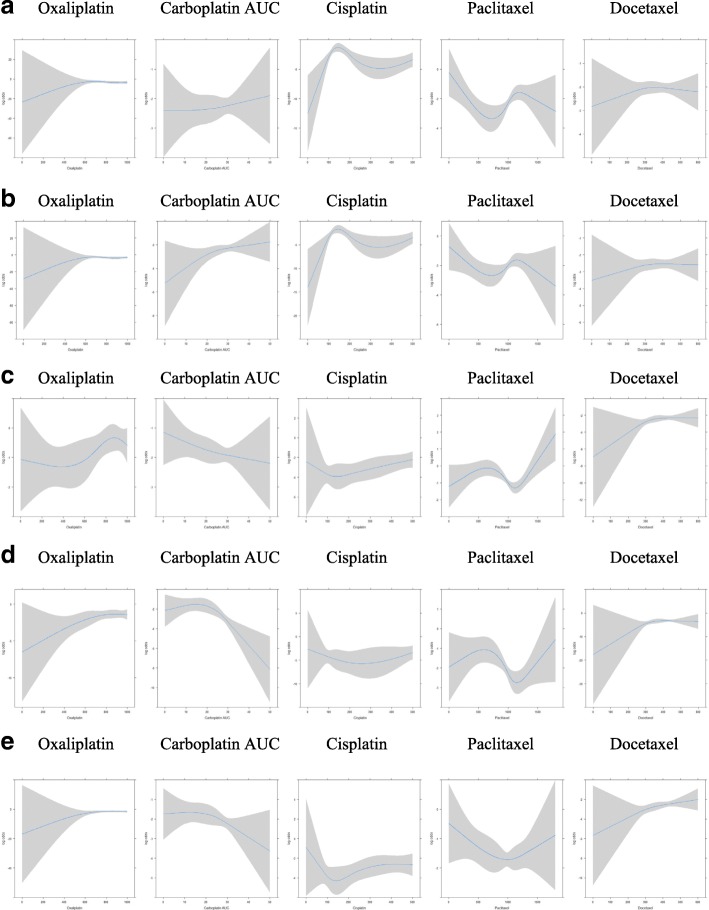
**a**. Dose-response relationship related to NCI CTCAE-motor dysfunction scale with oxaliplatin, carboplatin (AUC), cisplatin, paclitaxel and docetaxel respectively **b**. Dose-response relationship related to NCI CTCAE-sensory dysfunction scale with oxaliplatin, carboplatin (AUC), cisplatin, paclitaxel and docetaxel respectively **c**. Dose-response relationship related to the WHO neuropathy scale with oxaliplatin, carboplatin (AUC), cisplatin, paclitaxel and docetaxel respectively 6 **d**. Dose-response relationship related to cotton wool assessment with oxaliplatin, carboplatin (AUC), cisplatin, paclitaxel and docetaxel respectively **e**. Dose-response relationship related to monofilament assessment with oxaliplatin, carboplatin (AUC), cisplatin, paclitaxel and docetaxel respectively

Figures 6a-e show the dose-response relationship in different chemotherapy agents. No relationship was found for CIPN increases relative to the agent’s cumulative dose (Table 4). In most agents CIPN was established early in the treatment and continued to increase or remained relatively stable irrespective of the cumulative dose. This analysis was followed by a segmented regression for each chemotherapy agent, and again this showed no cumulative dose relationship with CIPN, with the exception of cisplatin-based regimens (=362 observations). In the latter, the turning point for motor CIPN (using CTCAE) was a dose of 249 mg/m2 (*p* < 0.001) and for sensory CIPN was 234 mg/m2 (*p* < 0.05), with lower cumulative dose than the turning point being linked with lower CIPN, and higher cumulative dose than the turning point not being associated with any higher CIPN.

**Table 4:** Odds ratios of the increased risk of developing neuropathy per 100 mg unit increase (or 1 AUC for carboplatin) of chemotherapy dose (generalized estimating equation)

	Abnormal				
Drug	NCI CTCAE motor	NCI CTCAE sensory	WHO CIPN	Cotton wool	Monofilament
Oxaliplatin-based regimens (*n* = 27, observations = 196)	1.15 (0.90, 1.47)	1.25 (0.94, 1.67)	1.13 (0.95, 1.34)	1.32* (1.06, 1.64)	1.42* (1.06, 1.89)
Carboplatin AUC (in 1 unit) (*n* = 88, observations = 657)	1.03 (0.98, 1.08)	1.06* (1.01, 1.10)	0.98 (0.93, 1.03)	0.93* (0.88, 0.99)	0.96 (0.92, 1.01)
Cisplatin-based regimens (*n* = 70, observations = 362)	0.89 (0.55, 1.46)	0.90 (0.55, 1.49)	1.43 (0.94, 2.19)	1.28 (0.57, 2.91)	1.37 (0.86, 2.17)
Paclitaxel-based regimens (*n* = 83, observations = 621)	1.02 (0.83, 1.25)	1.01 (0.85, 1.20)	1.01 (0.92, 1.11)	0.91 (0.80, 1.04)	0.94 (0.84, 1.06)
Docetaxel-based regimens (*n* = 151, observations = 1167)	0.96 (0.69, 1.32)	1.03 (0.69, 1.52)	1.36 (0.91, 2.04)	2.12 (0.93, 4.81)	1.47 (0.96, 2.25)

*Significant at 5% level

### Correlations between scales

Among the four clinician-based diagnostic scales, correlations were moderate to low at best. Often scales were not correlated with each other, and they were showing somewhat stronger correlations in later assessments, when CIPN was more well-established. The WHO criterion had correlations of r_s_ = 0.21–0.37 (p < 0.001) with the other scales at baseline and highest at 9-month (r_s_ = 0.44–0.65, *p* < 0.001) and 12-month (r_s_ = 0.40–0.59, *p* < 0.001) assessment. The WHO criterion was correlated more with the (sensory) monofilament assessment (r_s_ = 0.21–0.68, *p* < 0.001) and had low to moderate correlations (r_s_ = 0.15, *p* < 0.05 - r_s_ = 0.48, *p* < 0.001) with the sensory CIPN CTCAE.

### Nerve conduction study (NCS)

**Fig. 7:**
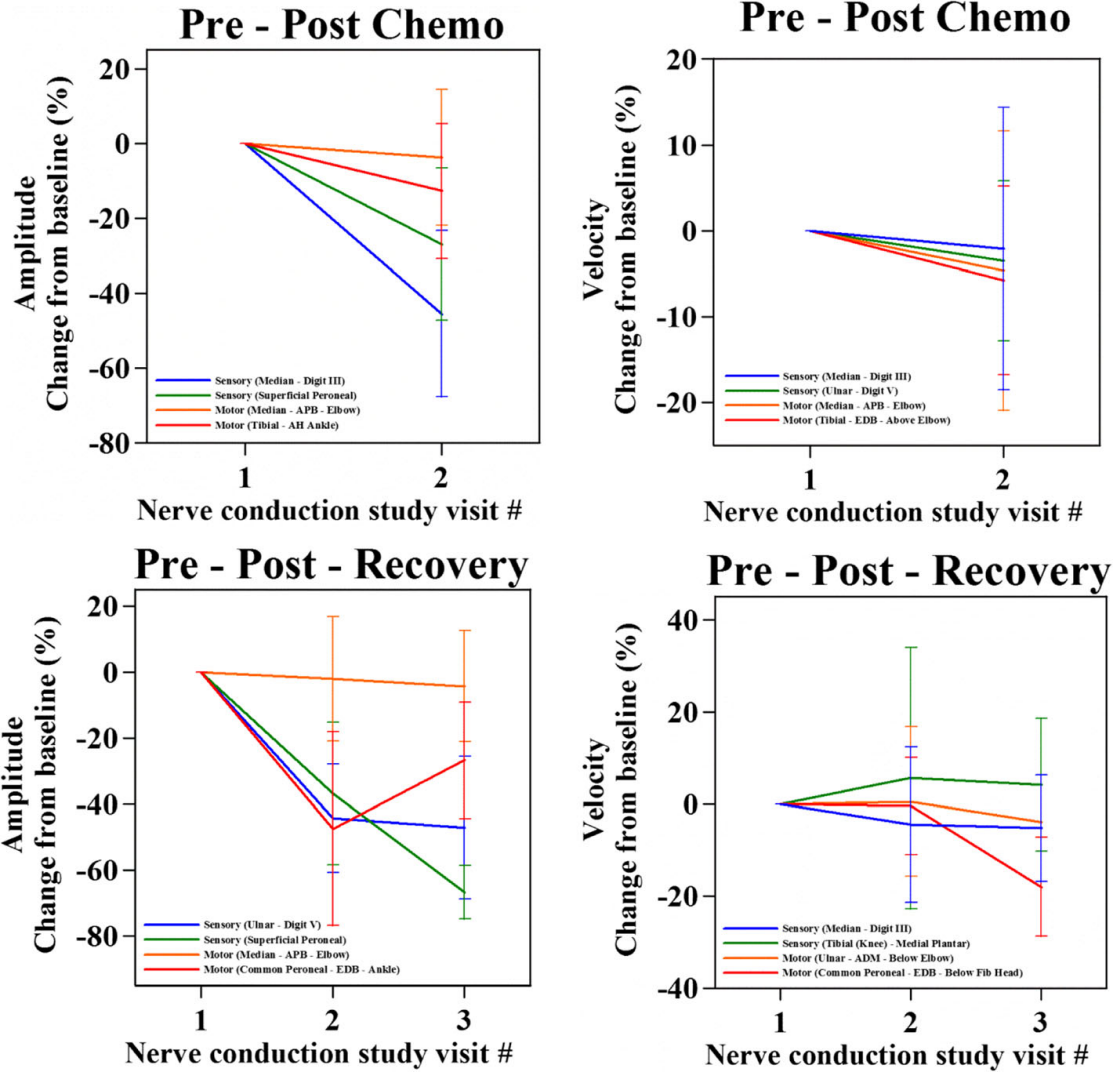
Changes observed in chemotherapy-induced peripheral neuropathy through Nerve Conduction Studies. The graph values correspond to the average of percentage differences to baseline (pre-NCS) of post-NCS and recovery-NCS

Patients treated with taxane chemotherapy showed a substantial decrease in SNAP (sensory) amplitudes at NCS_end_ (35.52 ± 4.48% lesser compared to NCS_baseline_). Specifically, the SNAP amplitudes of the upper limbs were affected nearly twice as much as the lower limbs (42.79 ± 3.90% and 26.71 ± 8.45% lower than NCS_baseline_, respectively). In contrast, the cMAP (motor) amplitudes of the lower limbs showed substantial decrease at NCS_end_, compared to the upper limbs (17.42 ± 6.51% and 1.87 ± 2.23% lower than NCS_baseline_, respectively). Compared to the amplitudes, the sensory and motor velocities displayed a minor decrease at NCS_end_ (1.54 ± 2.52% and 2.05 ± 1.63% lower than NCS_baseline_, respectively) (Fig. 7).

At 3 months post chemotherapy, i.e. NCS_3m_, patients continued to demonstrate a large decrease in SNAP amplitudes (a further decrease of 7.79 ± 7.05% lower than NCS_end_). It is to be noted that the upper limbs (4.85 ± 8.15% lower than NCS_end_) had better recovery compared to the lower limbs (18.33 ± 13.97% lower than NCS_end_). On the other hand, the SNAP velocities had negligible improvement (0.69 ± 2.78% lower than NCS_end_). The cMAP amplitudes, showed a recovery at NCS_3m_ (4.34 ± 3.05% higher than NCS_end_). The upper limbs did not exhibit much change (0.48 ± 2.03% lower than NCS_end_), while the lower limbs showed a considerable increase in NCS value (39.41 ± 16.28% higher than NCS_end_). Similar to the NCS_3m_ trend of the SNAP amplitudes, the motor velocities suffered a large deterioration (8.24 ± 3.91% lower than NCS_end_), with the upper limbs (6.54 ± 3.99% lower than NCS_end_) better than the lower limbs (20.28 ± 14.07% compared to lower than NCS_end_) (Fig. 7).

## Discussion

The current study is the largest and one of a few longitudinal assessments of CIPN (2399 observations) using a heterogeneous population that increases the generalizability of the results. It showed that prevalence was significantly different from measure to measure used, patient reported outcome measures (through quality of life scales) consistently showed much higher CIPN rates than clinician-based or objective measures, and with highest rates in patients receiving taxanes (primarily paclitaxel). Cumulative dose was not linked with higher CIPN prevalence, but time since starting chemotherapy was. Correlations among scales were generally low, although these increased in the later assessments primarily among sensory neuropathy assessments. NCS data confirmed the sensory and motor impairment of participants.

This study confirms that there is wide variation of CIPN prevalence between scales. This is supported by a meta-analysis of CIPN prevalence studies, where confidence intervals were high (i.e. at 3 months CIs = 37–84%) [3]. CIPN prevalence is reported in the literature to be high, from 54 to 73% in some studies [31–33] but often studies are using patient-reported outcome measures to assess prevalence, where CIPN prevalence is not the primary objective. Even in our study, when patient-reported outcome measures were used, prevalence doubled or tripled from that identified by other measures. It remains to be seen if such measures are appropriate diagnostic tools, as they also include items related to autonomic changes; such changes may have pre-existed the chemotherapy and it is unclear if they represent true CIPN-related changes or they are unrelated symptoms, particularly as many of the reporting studies are cross-sectional in nature. In our data, about one-quarter of the patients reported symptoms in the EORTC quality of life CIPN subscales at baseline, before they received any chemotherapy (Table 3). Nevertheless, other studies show similar results with our study including a one-year cumulative prevalence of 28.7% [34], 23% in a sample of patients receiving docetaxel [7] and 14.1% at 1 year [35]. Thus, it is clear that if we focus on prevalence rates that do not derive from patient-reported outcome measures, then prevalence is generally lower to about 15–25% of patients, the severity is generally not high, and that patient-reported outcome measures may overestimate CIPN prevalence as they include symptoms that may have pre-existed the chemotherapy. If patient-reported outcome measures, which are generally used to assess the ‘impact’ of a symptom on patients’ lives rather than diagnose a condition, are used to show CIPN prevalence, it has to focus more clearly on symptomatology that has developed after the use of chemotherapy, and exclude those that had similar symptoms before the chemotherapy. The latter number we have seen in our study was as high as 21.2%. Hence, cross-sectional assessments of CIPN using such patient-reported outcome measures can overestimate its prevalence. Irrespective of prevalence rates, however, the impact of the presence of such symptoms is significant [4, 6–10, 36] and every effort should be made to improve the patient symptom experience.

The issue of reliable and valid measurement of CIPN is fundamental, and based on the (relatively low) correlations between scales shown in this study, it may be that each scale measures a different phenomenon or the scales used in this study were not all sensitive enough to detect CIPN. Which scale from the available ones is best for clinical assessment remains to be identified, and perhaps there is a need for a combination of scales to be used [37]. In our study, the cotton wool assessment led to the lowest ‘pick up’ rates and at a later time from all other scales suggesting this may not be an appropriate and sensitive enough test. The WHO criterion and the monofilament were highly correlated, suggesting that the former measures more sensory changes. However, the WHO criterion is minimally used in current practice. Abnormalities in vibration and monofilament examinations are associated with abnormal sural nerve amplitudes [38] and hence monofilament may be a useful method in identifying CIPN. The CTCAE is the only scale that assesses separately motor and sensory problems (alongside quality of life scales/ patient-reported outcome measures); while this is an important dimension, in our study the CTCAE identified less patients with CIPN than other scales, suggesting that it may not have high sensitivity. Generally speaking, CIPN is not assessed properly and we should focus more on improving its assessment [6].

A recent review [39] suggests that CIPN is a predominantly sensory symptom with pain; however, in our study we obsered a considerable prevalence of motor neuropathy, particularly in the docetaxel subgroup, across assessments as well as paclitaxel and paclitaxel/carboplatin in the later assessments. This finding should be interpreted with caution as the only motor neuropathy-specific scale we used was the CTCAE item, and the literature suggests that the CTACE motor item overestimates its occurrence, possibly as a result of confounding factors [40] although NCS data confirms motor impairment in this sample (suggested by the decreased motor nerve amplitude in our sample). Also, it is unclear whether the motor dysfunction observed is true motor neuropathy or fine motor impairments secondary to sensory loss, which is difficult to distinguish when ‘blunt’ instruments such as the CTC are used. Motor dysfunction has been shown in a small-scale study, where decreased superexcitability of motor axons was reported [41]. Furthermore, a neurophysiological study in children receiving vincristine showed impaired myotatic reflexes and motor neural impairment [42] and a larger study in oxaliplatin-treated patients found no nerve dysfunction before the initiation of chemotherapy [43] while others studies (ie. ref. [38]) have focused on sensory subclinical changes only. NCS focusing on motor nerves, in addition to sensory nerves, could highlight if the above comment is correct, and Kandula et al. [44] review the diagnostic role of NCS in CIPN. It may be that there is a general impression that CIPN is mainly sensory impairment as most of the studies in the past assessed sensory problems, with little assessment of motor problems. Motor symptoms may also be hidden under ‘fatigue’ too, and ‘feeling weak’ may be interpreted as tired rather than myopathy. Also, it is common in clinical practice to only ask patients if they have any numbness or tingling in the hands/feet, again focusing only on sensory symptoms. There is a need to focus more concretely on the assessment of motor symptoms in the future, as they are also linked with significant impact in daily activities, and define CIPN more broadly as a motor and sensory impairment accompanied by autonomic system manifestations.

Furthermore, pain was not a major issue and occurred in less than one-quarter of the participants, whereas tingling and numbness were most prevalent in more than half the sample. Pain may be more prevalent in oxaliplatin [45, 46] or paclitaxel-based chemotherapy but it seems to be less common in other taxane- or platinum-based chemotherapies [47]. Whether autonomic symptoms are the result of CIPN or symptoms reflecting other pre-existing conditions before chemotherapy treatment is not clear as yet, and more prospective work needs to be done in this area too. Also, many other symptoms were reported by patients at different degrees in each type of chemotherapy suggesting that CIPN is not the same symptom across taxanes and platins. This is an important finding to consider particularly when we assess patients or when therapeutic trials for CIPN (in terms of primary outcome) are planned.

At the 12-month assessment, a significant number (around 8% through objective and physician-based scales or 25–30% based on the patient-reported outcome measure) remained with CIPN symptoms, similar or somewhat lower to other studies in the past [7, 34, 35, 48]. As CIPN has been seen in patients even 11–12 years after chemotherapy [10, 49], it seems that this group of patients may experience chronic and long-lasting CIPN.

Cumulative dose of the chemotherapy was not a predictive factor for CIPN, against the currently held belief, in any of the chemotherapy regimens we assessed. An increasing body of literature shows similar results [31, 50]. This brings into question the current practice [51, 52] of dose reductions or chemotherapy discontinuation in patients experiencing CIPN. This finding, alongside with the key factor of time since chemotherapy, suggests that from the moment CIPN is experienced, it will continue to increase for the next few months and carry its course before we see any noticeable decreases after 6 months, irrespective of dose of chemotherapy [53], and patients may continue developing CIPN over time even if doses are reduced [54]. Whether treatment discontinuation or dose reductions have a real impact on decreasing CIPN should be assessed in future research.

Limitations of the study include the decrease in the sample over cycles (many patients did not complete more than 3–4 cycles or were ill/dead, giving significant missing data particularly in the 5th–6th cycle data. Some chemotherapy protocols also had a relatively small sample size. Where numbers were small, results should be interpreted with caution. Interrater reliability of the assessments may be an issue, although specific training and a protocol guide was provided to all assessors. The sample from Caucasians was also small compared to the Chinese and other Asian sample included. Some of the assessments used (i.e. WHO criterion; cotton wool) have not been rigorously evaluated as assessment methods in the CIPN context) and hence interpretation of results from these scales should be cautious. Cotton wool test and 10 g monofilament can detect sensory impairment, but they are unable to capture deep sensory impairment. This is typical of platinum drugs neuropathy and might partly explain why they are performing less efficiently than tuning fork examination in comparison with literature data.

## Conclusions

This study maps the development and progression of CIPN in patients receiving taxane- and platinum-based chemotherapy, showing distinct CIPN profiles. It shows a lower level of CIPN than previously reported with significant differences among different chemotherapy protocols and scales used to measure CIPN. It brings into question the sensitivity and/or appropriateness of scales currently used to measure CIPN. Nevertheless, CIPN is a clinical problem present even 1 year after treatments and needs careful clinical attention. Practice-important findings include that early CIPN predicts CIPN in subsequent cycles, and this is the case irrespective of cumulative dose of chemotherapy. More work is needed in ascertaining best assessment methods for clinical practice and the findings call for a re-think of current clinical practices.
